# A young patient with lower limb Ewing’s sarcoma experienced a cerebral infarction: A care-compliant case report

**DOI:** 10.1097/MD.0000000000047008

**Published:** 2026-01-09

**Authors:** Si-Wei Zhang, Dong-Xue Cao

**Affiliations:** a Department of Emergency Medicine, Xi’an Central Hospital, Xi’an, China.

**Keywords:** cerebral infarction, Ewing sarcoma, Trousseau syndrome

## Abstract

**Rationale:**

Ewing sarcoma (ES) is a rare bone tumor that most often occurs in adolescents and young adults and is a malignant tumor that mainly affects, bones and soft tissues.^[1]^ Trousseau syndrome refers to a series of blood clot events in veins and arteries that occur during malignant tumor progression due to problems with blood clotting and breakdown. It includes clinical manifestations such as pulmonary embolism, arterial thrombosis, deep vein thrombosis, and nonbacterial thrombotic endocarditis. Acute cerebral infarction can also occur as part of Trousseau’s syndrome. Due to the rarity of ES and the lack of information on late-stage patients, there are reports of ES leading to acute cerebral infarction.

**Patient concerns:**

A 41-year-old male patient with lower limb osteosarcoma suddenly experienced a cerebral infarction.

**Diagnoses:**

Lower limb juvenile sarcoma combined with Trousseau syndrome, cerebral infarction.

**Interventions:**

An emergency cerebral angiography, mechanical thrombectomy, and percutaneous superselective arteriography.

**Outcomes:**

The patient was conscious, spoke fluently, and had grade IV muscle strength in the left upper limb. The left lower limb had limited movement because of the tumor, but basic activities had returned to how they were before the illness. The patient is currently undergoing further treatment for left lower limb osteosarcoma.

**Lessons:**

This reports a case of lower extremity elephantiasis nostras combined with Trousseau syndrome that was complicated by acute cerebral infarction. Clinically, acute cerebral infarction resulting from ES is rare. This case report aims to increase clinicians’ awareness of this disease and enhance diagnostic accuracy. This article presents only a single case report, with certain limitations, and more cases need to be observed in clinical practice to inform clinical diagnosis and treatment.

## 1. Introduction

Ewing sarcoma (ES) is a rare bone tumor that most often occurs in adolescents and young adults and is a malignant tumor that mainly affects, bones and soft tissues^[1]^. Trousseau syndrome refers to a series of blood clot events in veins and arteries that occur during malignant tumor progression due to problems with blood clotting and breakdown. It includes clinical manifestations such as pulmonary embolism, arterial thrombosis, deep vein thrombosis, and nonbacterial thrombotic endocarditis. Acute cerebral infarction can also occur as part of Trousseau’s syndrome. Due to the rarity of ES and the lack of information on late-stage patients, there are reports of ES leading to acute cerebral infarction.

## 2. Case report

The patient: a 41-year-old male, was admitted to the emergency department of Xi’an Central Hospital on December 4, 2024, at 9:40. The patient experienced ringing in the ears pressure discomfort in the back of the head without obvious triggers 30 minutes prior to admission, followed by slurred speech and dizziness. A few minutes later, the patient became unresponsive, had rapid breathing, and exhibited seizures in the left upper limb. There was no vomiting, fever, or incontinence observed. He was transported to our hospital’s emergency department in an ambulance. The patient was diagnosed with left lower limb synovial sarcoma 18 months previously and underwent chemotherapy and local surgery without amputation. Over the past 3 months, he experienced worsening swelling and pain in the left lower limb, accompanied by skin ulceration and blackening of the left knee. There was no significant family history or allergy history.

**Figure 1.**
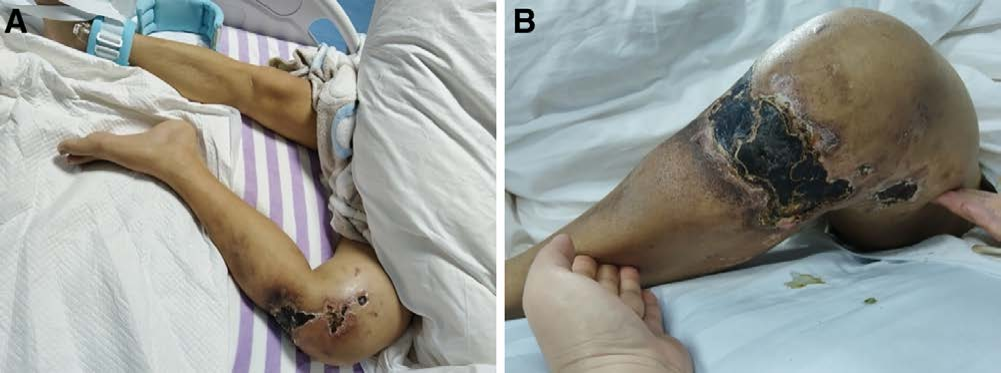
Condition of the tumor lesion in the patient’s left lower limb. (A) Comparison of the affected limb with the normal limb. (B) The affected tumor lesion area shows blackened and ulcerated skin surface, accompanied by exudate secretion.

Upon admission, a physical examination revealed a temperature of 36.3℃ pulse of 130 beats/min, respiration of 23 breaths/min, blood pressure of 156/105 mm Hg, and SO_2_ of 98%. Breath sounds were coarse bilaterally and no abnormal lung sounds were heard. The heart rate was 130 beats/min, with regular rhythm, and heart sounds were audible with no murmurs. The abdomen was soft with no tenderness or rebound tenderness, and bowel sounds were 3 to 4 times/min. Tumor tissue was visible in the left knee, with a 10 cm × 5 cm area of damage on the surface, showing a black scab with discharge (Fig. 1A, B). The left knee joint exhibited limited movement and tenderness. There was no edema in either lower limb. Neurological examination: gross olfactory testing was not responsive; gross visual testing was not responsive; visual fields were not responsive; the fundus looked normal; there was no ptosis in both eyelids, and both eyeballs moved freely in all directions; pupils were unequal, left 5 mm with sluggish light reflex, right 3 mm with sensitive light reflex; bilateraltemporalis and masseter muscles were symmetrical with no atrophy, and mouth opening was not participating; facial touch, pain, and temperature sensation were not responsive on both sides; corneal reflex was normal, and jaw reflex was normal. Bilateral forehead lines were symmetrical, nasolabial folds were symmetrical, and there was no deviation of the mouth corners; blowing cheeks did not participate, and taste sensation in the anterior two-thirds of the tongue was not tested. Hearing was grossly unresponsive. Nystagmus was not observed in the eyeballs. Shoulder shrugging and neck turning were not observed, tongue protrusions were not included, we could not measure muscle strength in the trunk and limbs, with stiffness in the left upper limb and normal muscle tone in the other limbs, and there were no involuntary movements. The finger–nose, finger–finger, alternating, and heel–knee–shin tests did not participate, and Romberg’s sign did not participate. The superficial sensations in the trunk and limbs were unresponsive. The bilateral biceps, triceps, brachioradialis, knee, and Achilles reflexes were all normal and symmetrical, and abdominal and cremasteric reflexes were present, with no patellar or ankle clonus. Left Hoffmann’s, Babinski’s, Chaddock’s, Oppenheim’s, and Gordon’s signs were all positive. The neck was soft, with no resistance; Kernig’s sign and Brudzinski’s sign were negative. The skin scratch test results were negative.

**Figure 2.**
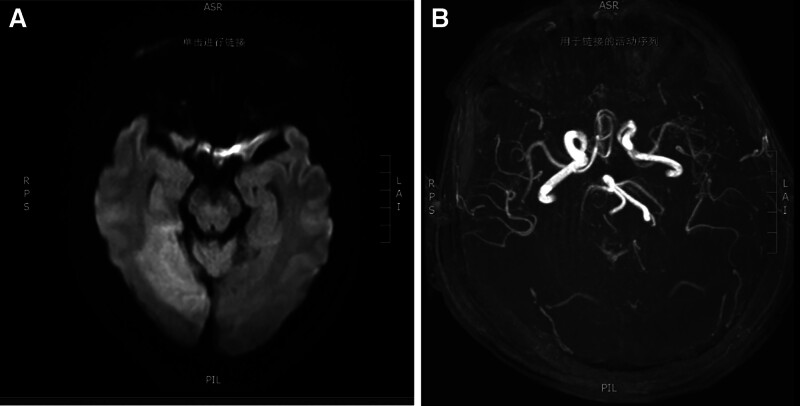
Patient’s cranial MR examination results: (A) acute infarction in the right occipital lobe, cuneus, thalamus, and deep temporal lobe; (B) right vertebral artery with tortuous course, occlusion of the right posterior cerebral artery, and localized stenosis of the right posterior inferior cerebellar artery lumen. MR = magnetic resonance.

**Figure 3.**
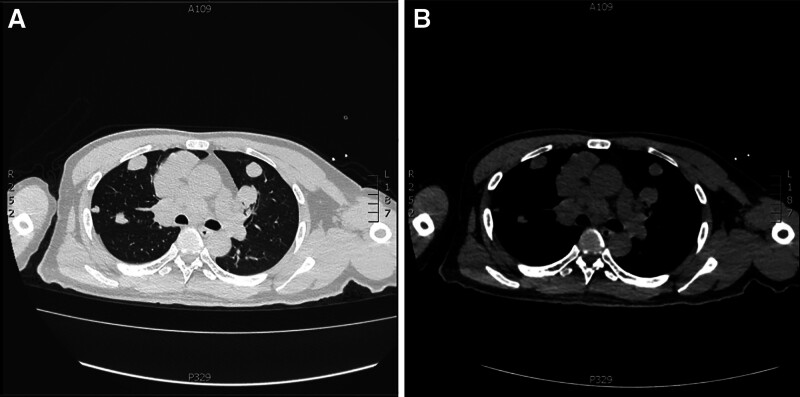
Patient chest CT examination results: (A) the lung window shows diffuse soft tissue lesions in both lungs combined with multiple enlarged lymph nodes in the mediastinum, some of which are fused, suggesting metastatic tumors (lung window); (B) in the mediastinal window at the same level, the lesions appear as solid soft tissue density. CT = computed tomography.

Auxiliary examination: electrocardiography showed sinus tachycardia and changes in the T wave. Head magnetic resonance imaging + magnetic resonance angiography + diffusion weighted imaging: acute infarction in the right occipital lobe, cuneus, thalamus, and deep temporal lobe (Fig. 2A); fresh punctate infarction in the left frontal lobe; the right vertebral artery has a curved course, occlusion of the right posterior cerebral artery, and local stenosis of the right posterior inferior cerebellar artery lumen (Fig. 2B); and multiple lacunar cerebral infractions in the bilateral basal ganglia, periventricular, subcortical frontal, and parietal lobes. Left lower limb computed tomography (CT): bone destruction of the left femur distal segment and proximal tibia with surrounding soft tissue mass; multiple dense shadows at the distal end of the left femur and proximal tibia, likely due to postoperative changes. A left knee joint effusion was observed. Chest-abdomen CT: there were diffuse soft tissue lesions in both lungs, along with multiple enlarged lymph nodes in the mediastinum, suggesting metastatic tumors (Fig. 3A, B); there was slight thickening at the junction of the left adrenal gland, and further examination recommended; the gallbladder was folded; there are a few fecal stones in the appendix; subcortical demyelination in the bilateral frontal regions. Complete blood count: white blood cell is 8.81 × 10^9^/L, neutrophilic granulocyte percentage is 77.50%, red blood corpuscle is 115 g/L, blood platelet is 572 × 10^9^/L, C-reactive protein is 224.87 mg/L. Biochemical results: Na^+^ 135 mmol/L, Cl^−^: 96.2 mmol/L, blood glucose 6.66 mmol/L, albumin 35.6 g/L, γ-glutamyl transferase 270.96 U/L, alkaline phosphatase 258 U/L, lactate dehydrogenase 2064 U/L, α-hydroxybutyrate dehydrogenase 1615 U/L, osmotic pressure 271 mOsm/L. The coagulation profile was as follows: prothrombin time 14.68 seconds, international normalized ratio 1.28, D-dimer 2.55 mg/L, fibrin (ogen) degradation products 9.66, fibrinogen 8.02 g/L.

Relevant examinations revealed that the patient had a large osteosarcoma lesion on the left lower limb. A CT scan of the left lower limb showed bone destruction in the lower part of the left femur and upper part of the tibia, accompanied by a surrounding soft tissue mass. Chest CT revealed multiple metastases in both lungs. Coagulation function tests suggested elevated D-dimer and fibrinogen levels, which indicated coagulopathy and increased fibrinolysis. Under these conditions, the patient suddenly experienced symptoms of altered consciousness and impaired limb movements. Cranial magnetic resonance imaging indicated acute cerebral infarction. This finding is consistent with Trousseau syndrome secondary to malignant tumors. The main clinical manifestation in this case was acute right posterior cerebral artery occlusion leading to extensive right cerebral infarction. Diagnosis: acute cerebral infarction (left parietal lobe and right cerebellum); right posterior cerebral artery occlusion; hypertension; and osteosarcoma of the left lower limb with multiple lung metastases.

## 3. Therapeutic intervention

**Figure 4.**
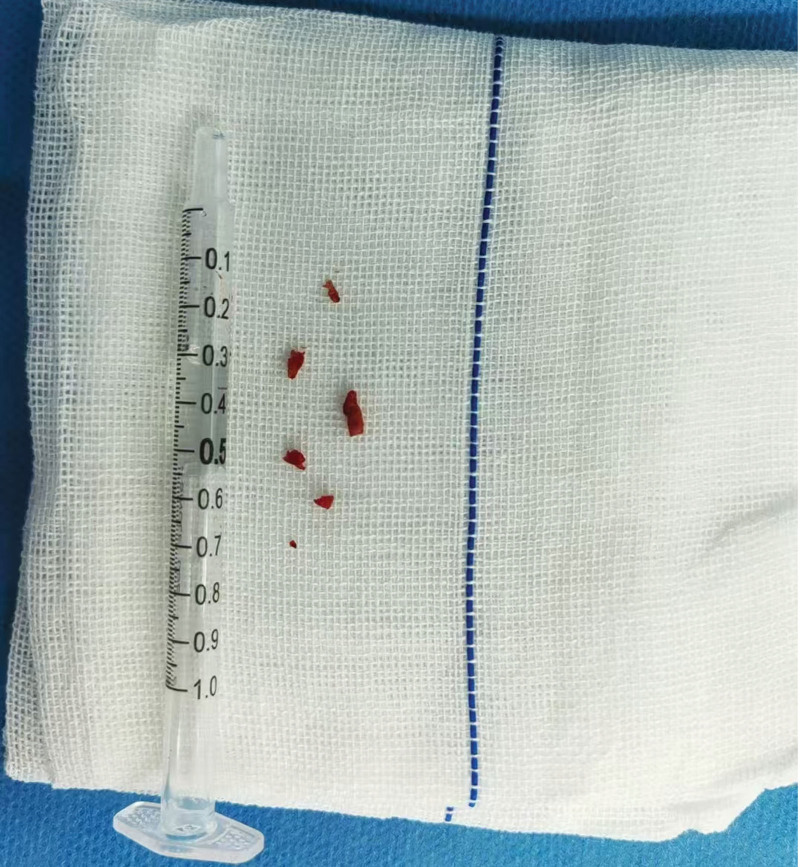
The thrombus removed from the patient.

The patient had no obvious contraindications for thrombolysis and emergency thrombolytic therapy was initiated. Based on the patient’s weight, 6.3 mg of alteplase was administered intravenously, followed by continuous infusion of 56.7 mg alteplase at a rate of 56.7 mg/h at 12:00. Thrombolysis was completed within 1 hour, during which the patient had no major side effects and there were no signs of bleeding from the skin, mucous membranes, urinary tract, or gastrointestinal tract. After thrombolysis, emergency cerebral angiography was performed, which revealed occlusion of the right posterior cerebral artery. Subsequently, an emergency procedure was performed under general anesthesia, which included cerebral angiography, aortic arch angiography, mechanical thrombectomy for acute occlusion of the P2 segment of the right posterior cerebral artery, and percutaneous superselective arteriography. The procedure proceeded smoothly, and the patient woke up after surgery, had the endotracheal tube removed, and was transferred to the intensive care unit for continued treatment (Fig. 4).

After thrombolysis combined with mechanical thrombectomy, the patient regained consciousness, was able to speak, showed no signs of aspiration when drinking, and had voluntary movements in the left upper limb. The patient was administered 40 mg atorvastatin calcium tablets once daily for lipid regulation, plaque stabilization, circulation improvement, and dehydration to reduce intracranial pressure, with dynamic observation of consciousness changes. The patient had a history of hypertension and was administered 100 mg of urapidil via continuous infusion to control blood pressure. The patient’s left lower limb osteosarcoma was accompanied by local skin damage with visible secretion, for which mupirocin was applied topically for dressing changes. Symptomatic treatments include correcting anemia, supplementing hematopoietic materials, and providing albumin. A complete blood count indicated a high percentage of neutrophils combined with elevated procalcitonin, for which 1.5 g of cefuroxime sodium was administered twice daily for anti-infection treatment. The patient was conscious, spoke fluently, and had grade IV muscle strength in the left upper limb. The left lower limb had limited movement because of the tumor, but basic activities had returned to how they were before the illness. The patient is currently undergoing further treatment for left lower limb osteosarcoma.

## 4. Discussion

ES is a type of small round cell sarcoma, and is a rare bone tumor most commonly found in adolescents and young adults, with an incidence of 0.3 per 1,00,000 people each year. ES is the third most common malignant bone tumor in humans, just behind osteosarcoma in children. The median age at diagnosis was 15 years, with male predominance (1.5:1). The primary tumor is usually found in the bones (long bone diaphysis, such as the femur, tibia, humerus, pelvis, chest wall, and spine), and mainly spreads through the bloodstream in later stages, with the lungs, bones, and bone marrow being the most common sites of metastasis.^[2]^ Approximately 1 in 4 patients already had metastases when they were diagnosed, with the lungs being the most common site (50%), followed by the bones (25%) and bone marrow (20%). ES is often associated with extensive necrosis.^[3]^

Trousseau syndrome was first noted in 1865 when Armand Trousseau found that patients with gastric cancer were prone to blood clots. Subsequently, the medical community started calling for a series of blood clot events that occurred due to abnormalities in coagulation and fibrinolysis mechanisms during the course of malignant tumors Trousseau syndrome. This includes symptoms such as pulmonary embolism, arterial thrombosis, deep vein thrombosis, and nonbacterial thrombotic endocarditis. Acute cerebral infarction is also a manifestation of Trousseau syndrome. Currently, there is little literature connecting ES to Trousseau syndrome. Trousseau syndrome is usually caused by thrombosis induced by malignant tumors. Therefore, in clinical cases of ES, although Trousseau syndrome is considered a relatively rare complication, an increasing number of studies suggest that ES may influence thrombosis through the following mechanisms. The mechanisms of thrombosis in patients with tumors are complex and varied, involving tumor cells, the microenvironment, coagulation factors, and immune responses. The association between tumors and thrombosis has also been widely studied. How tumor cells interact with platelets is the key to tumor growth and spread. Studies have shown that procoagulant factors released by tumor cells can clump together, forming microtumor thrombi that protect tumor cells from immune system attacks and promote the infiltration and metastasis of tumor cells.^[4,5]^ Additionally, the binding of tumor cells to platelets may affect the tumor microenvironment by altering platelet function, thereby promoting tumor growth and progression.^[6,7]^ Tissue factor is the main initiator of the extrinsic coagulation pathway, and high expression of tumor cells is closely related to the aggressiveness and metastatic potential of tumors.^[8,9]^ Abnormal expression of coagulation factors in tumors is also an important cause of thrombosis. Research has shown that immune cells and platelets in the tumor microenvironment are also involved in the abnormal expression of coagulation factors. For example, tumor-associated immune cells can promote the expression by releasing cytokines, further exacerbating the hypercoagulable state of the tumor.^[10]^ In the context of acute ischemic stroke immune cells play an important role in thrombosis in acute ischemic stroke. Recent studies have shown that immune cells not only participate in defense against pathogens but also directly contribute to thrombus formation, particularly monocytes and neutrophils, which promote thrombosis by releasing cytokines and forming neutrophil extracellular traps. Neutrophil extracellular traps capture pathogens and provide a scaffold that promotes platelet aggregation, thereby accelerating thrombus formation.^[11]^

Many patients with ES might have other risk factors (such as obesity, a history of thrombosis, prolonged bed rest, etc), which increase the risk of thrombosis along with the tumor. In this case, the tumor lesion was in the left lower limb, and the patient was bedridden for a long time, leading to a slowdown in local blood flow velocity. Reduced blood flow velocity favors the aggregation of platelets and coagulation factors, thereby increasing the risk of thrombosis. Trousseau syndrome in patients with ES may present with swelling, leg pain, and shortness of breath, and in this case, the patient presented primarily with acute stroke. As these symptoms are often linked to the tumor itself or are considered to be caused by brain metastases of the tumor, they may be overlooked. Therefore, doctors should look out for blood clots that could lead to different embolic diseases in ES patients. Additionally, it is important to conduct thrombosis risk assessment in patients with ES. For patients with high-risk factors, the use of anticoagulants should be considered for prevention. Furthermore, implementing early mobilization and physical therapy during chemotherapy or surgical procedures can help reduce the risk of thrombosis. As a malignant tumor, complications of Trousseau syndrome caused by ES require sufficient attention. Understanding the mechanisms of tumor-associated thrombosis can help doctors to take better preventive and treatment steps in managing patients. By enhancing awareness of this complication, it is possible to reduce the clinical risks for patients and improve treatment outcomes and quality of life.

## 5. Conclusion

ES is a rare malignant tumor that primarily occurs in bones and soft tissues. Reports of Trousseau syndrome associated with late-stage tumors are quite rare. In this case, the patient had an acute cerebrovascular embolism, which is uncommon in clinical practice. This case highlights the need to consider thrombotic events related to malignant tumors when diagnosing such patients. Getting an early and accurate diagnosis, along with prompt treatment, is really important for the patient’s recovery.

## Author contributions

**Conceptualization:** Dong-Xue Cao.

**Investigation:** Dong-Xue Cao.

**Methodology:** Si-Wei Zhang.

**Resources:** Dong-Xue Cao.

**Supervision:** Dong-Xue Cao.

**Writing – original draft:** Si-Wei Zhang.

**Writing – review & editing:** Si-Wei Zhang, Dong-Xue Cao.
